# Lessons Learned From the SoBeezy Program for Older Adults During the COVID-19 Pandemic: Experimentation and Evaluation

**DOI:** 10.2196/39185

**Published:** 2022-11-24

**Authors:** Marion Pech, Antoine Gbessemehlan, Lucile Dupuy, Hélène Sauzéon, Stéphane Lafitte, Philippe Bachelet, Hélène Amieva, Karine Pérès

**Affiliations:** 1 Bordeaux Population Health Research Center Inserm, UMR 1219 University of Bordeaux Bordeaux France; 2 The SoBeezy Project Pessac France; 3 National Institute for Research in Digital Science and Technology University of Bordeaux Bordeaux France; 4 Haut-Lévêque Cardiology Hospital University Hôpital of Bordeaux Pessac France; 5 University of Bordeaux Bordeaux France; 6 Centre d'Investigation Clinique Plurithématique de Bordeaux Pessac France

**Keywords:** voice assistance, social isolation, healthy aging, living in place, acceptability, technologies, digital divide, older adults, aging, elderly population, voice assistant, COVID-19

## Abstract

**Background:**

The SoBeezy program is an innovative intervention aimed at promoting and fostering healthy aging and aging in place by proposing to older adults concrete solutions to face daily life, tackle loneliness, promote social participation, and reduce the digital divide, thanks to a specific, easy-to-use voice assistant (the BeeVA smart display).

**Objective:**

This study aims to assess the acceptability of the SoBeezy program and its voice assistant and to identify potential areas of improvement.

**Methods:**

A 12-month experimentation of the program was deployed in real-life conditions among older adults living in the community in 4 pilot cities of France. Launched during the first lockdown of the COVID-19 crisis, this multisite study aimed to assess acceptability using questionnaires and interviews conducted at baseline and at the end of the experimentation. In addition, a series of meetings were conducted with SoBeezy staff members to obtain direct feedback from the ground.

**Results:**

In total, 109 older individuals were equipped with BeeVA to use the SoBeezy program; of these, 32 (29.4%) left the experimentation before its end and 69 (63.3%) completed the final questionnaires. In total, 335 interventions were conducted and 27 (39%) of the participants requested services, mainly for supportive calls and visits and assistance with shopping, transportation, and crafting-gardening. Of the whole sample, 52 (75%) considered BeeVA as a reassuring presence, and few persons (15/69, 22%) reported a negative opinion about the program. Among the participants, the voice assistant appeared easy to use (n=57, 82%) and useful (n=53, 77%). They also were positive about the BeeVA smart display and the SoBeezy intervention.

**Conclusions:**

This multisite study conducted in real-life conditions among more than 100 older adults living in the community provides enlightening results of the reality from the ground of digital tools designed for the aging population. The COVID-19 context appeared both as an opportunity, given the massive needs of the older adults during this crisis, and as limiting due to sanitary constraints. Nevertheless, the experimentation showed overall good acceptability of the voice assistant and a high level of satisfaction of the participants among those who really used the system and could be a way of improving the autonomy and well-being of older adults and their families. However, the findings also highlighted resistance to change and difficulties for the users to ask for help. The experimentation also emphasized levers for next deployments and future research. The next step will be the experimentation of the activity-sharing component that could not be tested due to the COVID-19 context.

## Introduction

The increase in life expectancy along with the massive arrival of baby boomers at retirement age have led to a major transformation of the age population structure. In 2040, 1 in 4 inhabitants will be 65 years old or over compared to 18% in 2013, and by 2070, the population aged 75 years or more would be twice as numerous as in 2013 (+7.8 million) [1]. In this context, healthy aging has become a major challenge for societies, as suggested by various plans, programs, and policy orientations worldwide [2-6]. Beyond the obvious health factors central to the healthy aging process, personal factors (eg, resilience abilities, self-esteem, personality traits) and environmental ones (eg, social support, living environment) also play a crucial role [7]. For instance, loneliness and social isolation, accentuated during the COVID-19 crisis, are growing at an impressive pace, particularly in the older population [8-11]. Approximately half of the adults aged 60 years and over are at risk of social isolation [12], and one-third will experience some degree of loneliness in later life [13,14]. It is now well established that loneliness and social isolation compromise living in place [15,16] and are risk factors for an unhealthy lifestyle (eg, sedentary lifestyle, poor diet, tobacco, alcohol), morbidity (eg, chronic conditions, such as cardiovascular disease, stroke, dementia, depression, anxiety, and disability), and mortality [17-19]. Therefore, to tackle social isolation and loneliness among older adults, numerous interventions are being developed and deployed [16], many of them involving technological devices.

Technology plays an increasingly central role in the ways we communicate. Indeed, we are witnessing a paradigm shift in communication, where face-to-face exchanges are no longer the only way to maintain interpersonal connection [20], thanks to information communication technology (ICT) [21,22]. There are 2 types of online services [23,24], social network services (SNS) and social online services (SOS). SNS were created first. They are online environments where users create a personal profile, build a network of personal connections [23,25], and can thus stay in touch with friends, family, and acquaintances (eg, Facebook) [26]. For instance, Neves et al [21] developed an age-adapted app on iPad that allows nursing home residents to stay in touch with their families by providing easy access to photos, audio recordings, videos, and messages. This app increased residents’ perceived levels of social interactions but only among people whose relatives were geographically distant and people who had a higher need to feel socially included, which drove them to adapt to technological constraints. Other SNS have been developed, such as Meeteetse [27], the ASTRA app [28], ShoddyPop, and PersonCard [29]. Following the advent of SNS, 2 types of social platforms arose, social commerce platforms (eg, eBay) and social solidarity platforms (eg, based on collaborative consumption platforms, such as Swaptree, Airbnb, Getaround, and Taskrabbit), both based on the trade and exchange of goods and services between people. Social solidarity platforms may be useful to older adults to help with daily activities and promote social participation [24]. A systematic review [30] identified social platforms dedicated to older persons that provide easy access to information sources and communication opportunities. These platforms enhance social connectivity and promote healthy lifestyles, including physical and cognitive activities (eg, games activities, such as the Wii console), a safer environment, and positive emotions. They include several functions, such as self-monitoring, calendars, photos, games, and online assistance [31-35]. Based on the sharing of personal services, which hold the potential to strengthen social integration and enable an independent lifestyle for older adults, Koene et al [24] developed a local, service-oriented collaborative consumption platform called “Bring Dich ein!”. This platform aims to facilitate social interactions across generations and peer-to-peer services. The platform was implemented in a participatory development process. In the pilot phase, usability was good, but in the absence of subsequent publication, we cannot know whether the promise has been kept [24].

To summarize, SNS target more loneliness, enabling the creation or maintenance of social interactions [23], whereas SOS support accessibility of services to compensate and help people with health problems, disabilities (eg, walking difficulties), and social isolation issues [24,36]. With advancing age, older adults may need to stay in touch with their family or friends and exchange services within a secure community. For these reasons, a few studies have tried to combine SNS and SOS to increase the appropriation of such technologies. For instance, Personal Reminder Information and Social Management (PRISM) is a software application [31] designed to support social connectivity, memory, knowledge about various topics, leisure activities, and access to resources. Boll and Brune [36] proposed a prototype platform providing an integrated online environment, in particular to help bridge the gap between older adults, and services from professionals as well as from other users. However, we lack robust data to assess the benefit of this combination. On the one hand, grouping services and social networks in a single platform seems helpful. On the other hand, the use of the device could be harder to understand (longer menus, more services with more complicated pathways) [37], potentially leading to a negative user experience, especially in older users [38].

When focusing on older users, the conception and implementation of online services must address several ergonomic issues, such as age-appropriate design (ie, ease of use), compatibility with the user’s needs (ie, usefulness), and technical issues, such as reliability [39] and privacy [23] concerns. Characteristics of the older users themselves, such as familiarity with technologies, adequate social support, cognitive abilities, or health status, also need to be considered since these factors can also influence technology use [39]. For instance, interfaces are often too complex, have too many options, and are not appropriate for “nontypical” users who suffer from sensory or cognitive impairments or do not have a technological background. Furthermore, the user interface should be as simple as possible, for example, by grouping similar items and functionalities to help users who have no experience in using this type of interface, by providing the users with only essential information, by increasing header and content sizes, and by giving the option to zoom in and out for people with visual impairments. These solutions could be easily implemented and do not presume drastic changes in the standard user interaction of SNS, such as Facebook [23]. Multimodality (multiple modes of interacting with a system) is also recommended for intuitive use. It provides an opportunity to the user to choose the best-adapted mode regarding their skills, abilities, habits, and wishes. These functionalities, such as text-to-speech, text input, speech commands, or other augmentative alternatives, could have a positive impact on device appropriation [23]. Several requirements and design rationales were deemed essential by older adults, such as intuitive interaction and navigation, a closed community, strong privacy policies, and community consciousness [24].

Technology may deeply modify the ways we communicate and could be relevant to tackle loneliness. However, isolated people are also less likely to use these types of devices due to lower skills, greater reactance, and lack of motivation and support from family [40]. In the current intensive process of world digitalization, there is an urgent need to create accessible, adaptable, and easy-to-use tools for all to reduce the associated risk of social exclusion of the older population.

In this context, the SoBeezy program has been developed to foster healthy aging at home by facilitating and improving older adults’ daily life [2]. The system proposes solutions to face the main difficulties encountered in daily life and fosters social participation by promoting community-based cooperation and the sharing of activities and experiences. The program relies on (1) an intelligent digital platform available on smartphones, tablets, and computers and also a voice assistant (BeeVA) specifically developed for people with a digital divide; (2) an extensive solidarity network that potentially relies on everyone's engagement through an intergenerational approach [41,42], where older people themselves are not only service receivers but also potential contributors; and (3) all the local partners and stakeholders available to cooperate (associations, social services of municipalities, health professionals, home care services, and all relevant local partners, such as artisans). The SoBeezy program is organized as a hub and connects all the territory's resources to provide the best solution to meet the user’s needs. The program has been implemented for 12 months, specifically targeting older adults living alone or suffering from loneliness. The objectives of this study were (1) to assess the usage, service satisfaction, acceptability of BeeVA and, more generally, the SoBeezy program and (2) to identify the potential amendments that should be provided to improve the system.

## Methods

### A 12-Month Experimentation in Real-Life Conditions Among Older Adults Living in the Community

As previously published [2], the initial protocol of the SoBeezy program planned before-after analyses and a comparative approach with a control group to assess the impact and effectiveness on healthy aging, technical usage, mechanisms of intervention, and conditions of transferability and scalability. However, due to the particular context of the COVID-19 pandemic and given the massive needs of the older population at that time, we decided to anticipate the launch of the program and to prioritize the deployment of the device and the assistance given to older adults. Consequently, the evaluative research could not be implemented, as planned, and had to be adapted to this extraordinary context: removal of several services and activities, impossibility to recruit a control group in the pandemic context, and baseline data collection restricted to the bare minimum (as detailed later).

**Figure 1:**
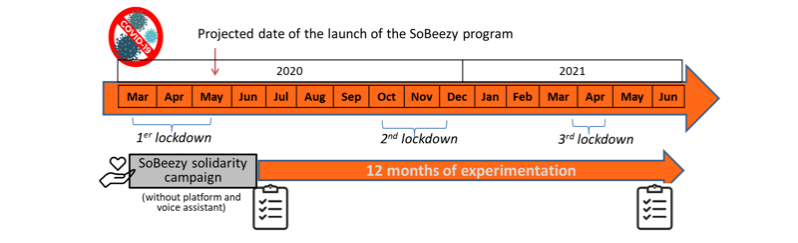
Representation of the SoBeezy experimentation in the general population.

**Figure 2:**
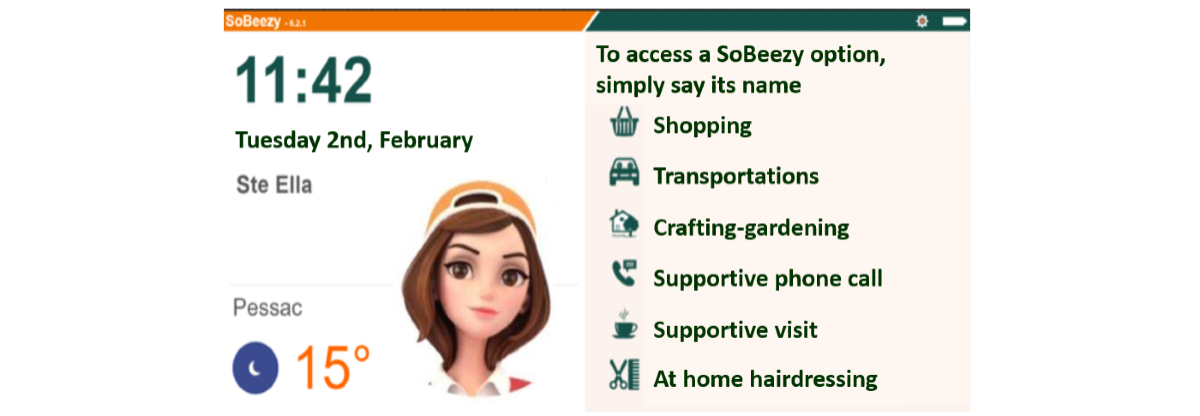
The BeeVA home page.

**Figure 3:**
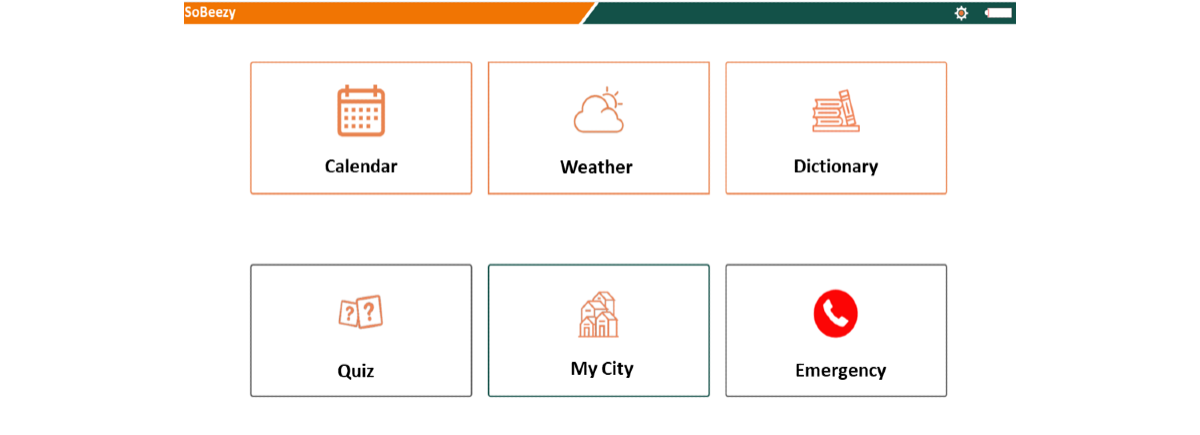
Options section.

Initially scheduled in May 2020, the launch of the program was anticipated with a solidarity campaign during the first lockdown (supportive calls and assistance with shopping, transportation, dog walking without the SoBeezy technology) in April 2020 to respond to the massive needs generated by the COVID-19 crisis (Figure 1). Then from July, these services were extended to crafting-gardening, supportive visits, and at-home hairdressing and were made available on the SoBeezy platform and the BeeVA smart display (Hello 10 Archos). However, the program has only been partially deployed due to the restrictive barrier measures. Indeed, the activity-sharing component could not be analyzed and the assistance in daily living was only limited to the essentials (Figure 2). BeeVA also proposed several options, such as weather forecast, radio, a digital calendar, emergency numbers, games, and city news (Figure 3).

The experimentation started in July 2020 with the installation of BeeVA in the participants' homes as soon as it was allowed by sanitary measures (installations staggered over the first 6 months) and ended in June 2021. It took place in 4 pilot cities (2 urban cities, Pessac and Saint Jean de Luz, and 2 rural cities, St Yrieix la Perche and Langon) in southwest France, with a close partnership with the municipalities. The participants were recruited among older adults supported by the SoBeezy solidarity campaign but also with the support of local social services (municipalities), health professionals, local associations, a private social protection agency (AG2R La Mondiale), and communication campaigns (press, radio, social media). The eligibility criteria were being 50 years and over, living in 1 of the 4 pilot cities, living in an area with sufficient access to high-speed internet service, being free of severe visual or hearing impairments, being free of moderate-to-severe cognitive impairment, and being a French speaker (for better voice recognition by BeeVA). All participants were equipped (free of charge) with BeeVA and an internet connection (a 4G Wi-Fi device).

Each person potentially interested in participating received the first visit at home for a detailed presentation of the program and BeeVA. A second visit was scheduled to install the device and propose the first user training if the person was still interested. A user manual provided instructions, including instructions in the case of technical problems as well as a specific hotline phone number. A few days later, a phone call aimed at ensuring the correct handling and use of the device. Other training/coaching visits were conducted as many times as necessary, and regular follow-up phone calls were made.

### Evaluation of the Experimentation

To assess the acceptability of the program and of BeeVA, questionnaires and interviews were administered at baseline and after 12 months (Figure 1). Due to the COVID-19 context, evaluations were conducted by phone to respect the barrier measures, as recommended.

#### General Individual Data

Information about age, gender, living status (living alone vs not living alone), city, beneficiary of home care services, member of associations, self-reported global health, and comfort level with online technologies was collected.

#### Acceptability of BeeVA

The number of requested services and options used by the participants was collected automatically by the system (log data). Usage frequency (number of interactions per month) was also recorded. At the final assessment, the participants were invited to answer 17 questions (Multimedia Appendix 1). As recommended by Chen and Chan [39], we distinguished the following 5 dimensions: perceived usefulness (4 items), usage behavior (4 items relative to the options used), ease of use (3 items), reliability (1 item), and appreciation of the design and ergonomics of the device (5 items).

#### Service Satisfaction of the SoBeezy Program

In total, 5 items were used (Multimedia Appendix 1): satisfaction regarding the services provided by SoBeezy, delay of answers and intervention, quality of the relationships with SoBeezy staff members, volunteers and professionals, and communication preferences with these 3 contributors, with 3 answer modalities (rather yes/rather no/never used).

#### Global Perception of the SoBeezy Intervention

We combined 1 item related to BeeVA (“Is the voice assistant a useful tool?”) and 1 related to the services (“Are you satisfied with the services provided?”). We obtained 3 main opinions about SoBeezy: (1) 2 positive answers defined a positive opinion, (2) only 1 positive answer defined a mixed opinion, and (3) negative answers or no usage defined a negative opinion.

#### Improvement Tracks

In the perspective of improving the system, the participants were also invited to assess the usefulness/interest of 18 new possible options and features on a scale ranging from 1 (not interested at all) to 7 (very interested). These options included photos and messages sent by the family, music, radio, audiobooks, games or an e-calendar, and medication reminders (for a detailed description of the options, see Multimedia Appendix 2). Finally, the activity-sharing (leisure, physical and cultural activities) component that could not be experimented upon due to the COVID-19 crisis was also proposed as a future option.

In addition, to collect direct feedback from the SoBeezy staff members (comprising volunteers and employees), a series of meetings were conducted at the end of the experimentation, with 4 main topics: the health crisis context, older users, technological aspects, and organizational challenges.

### Statistical Analysis

Statistical analyses were mainly descriptive and comparative. We reported means and SDs for continuous variables and frequencies for categorical variables. For comparative analyses, adequate statistical tests (chi-square and Fisher test) were performed. All analyses were performed using R version 4.1.2 (R Foundation for Statistical Computing).

### Ethical Considerations

All participants provided written informed consent to participate in the study. Data protection complies with European and French data protection regulations (GDPR and CNIL). Privacy and confidentiality protection was ensured by systematically conducted statistical analyses on de-identified data. The protocol and informed consent and assent forms were approved by the Comité d'Evaluation Ethique de l'INSERM (CEEI; Institutional Review Board [IRB] approval 2020-16/05). Finally, as compensation for their participation in the research, the smart display was offered to each participant.

## Results

### Sample Description

**Figure 4:**
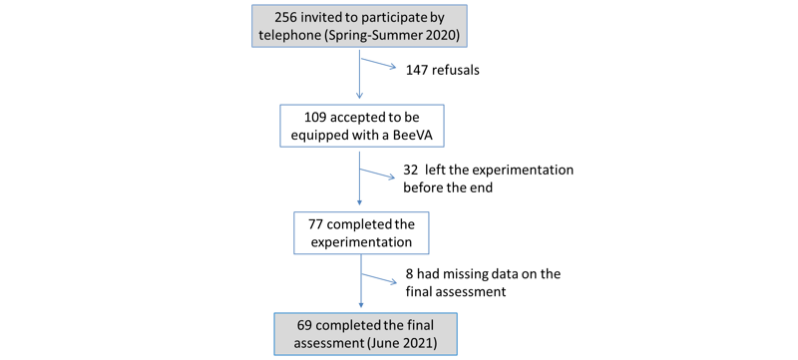
Flowchart of the description of the sample.

In total, 256 persons were invited to participate in the study by telephone, of which 109 (43% participation rate) accepted. Among them, 77 (71%) completed the experimentation, and 69 (90%) of them completed the final assessment conducted at the end of the study in June 2021 (Figure 4). In total, 109 participants were equipped with BeeVA. The mean age was 81.2 years (SD 8.6), 86 (78.9%) were women, 66 (60.6%) lived in Pessac City, and 44 (55.7%) reported alteration in general health (eg, walking difficulty). See Table 1 for details.

Among the 109 participants, 32 (29.4%) requested the device to be uninstalled before the end of the study, after 2.8 months (SD 2.5) of use, on average. These participants were more likely to be older (mean 83.2 years, SD 7.9 years) compared to others (mean 80.4 years, SD 8.8 years), women (n=27, 84%, vs n=59, 77%), and tended to use less often the device and services.

Among the 109 participants, 47 (43.1%) used BeeVA for 8-12 months (the device installation being staggered over the first 6 months and 32 participants leaving the study prematurely). Of the 69 participants who completed the experimentation, 41 (59%) used it beyond 8 months.

Using the general information collected at baseline, we proposed a description of the characteristics of the completers and noncompleters in Table 1. Among the completers, almost 75% (n=52) were living alone, one-third (n=23, 33%) benefitted from home care services, and half (n=35, 51%) were members of an association (Table 1). Concerning the general use of technologies, the smartphone was the most frequently used device (n=39, 57%, using it regularly), followed by the computer (n=30, 21%), while only 9 (13%) participants used a digital tablet. In addition, 35 (56%) participants estimated their comfort level using technology as poor, with a higher frequency among the oldest participants, those living alone, and those with good global health/mobility (Table 1).

To identify the main reasons for ceasing participation, we conducted a qualitative analysis of the individual files (no information available for 5, 16%, of 32 participants). The 3 main reasons were technical problems (internet, breakdown, or difficulty using the BeeVA; n=8, 25%), followed by health problems (n=5, 16%), and no need of services (n=5, 16%).

**Table 1:** Description and comparison of samples of completers (n=69) and noncompleters (n=40).

Characteristics		Completers (evaluation sample; n=69)	Noncompleters (n=40)
Age (years), mean (SD)		80.2 (8.9)	82.9 (7.8)
Women, n (%)		53 (77)	33 (83)
**Pilot city, n (%)**			
	Pessac (urban area)	42 (61)	24 (60)
	St Jean de Luz (urban area)	14 (20)	11 (28)
	St Yrieix la Perche (rural area)	8 (12)	2 (5)
	Langon (rural area)	5 (7)	3 (8)
Living alone^a^, n (%)		49 (71)	N/A^b^
Recipient of home care services^a^, n (%)		23 (33)	N/A
Member of an association^a^, n (%)		32 (46)	N/A
Not comfortable with online technologies^a^, n (%)		35 (56)	N/A
**Global health, n (%)**			
	No self-reported problems	29 (42)	6 (60)
	Walking difficulty	13 (19)	2 (20)
	Other self-reported health problems	27 (39)	2 (20)

^a^Data only available for participants seen at the final visit (n=69).

^b^N/A: not applicable.

### Usage and Satisfaction Regarding SoBeezy Services

Of the 69 participants who completed the experimentation, 27 (39%) used the services proposed by the platform at least once (Table 2). In total, 335 services were provided, 132 (39.4%) thanks to the solidarity campaign (before the technological deployment) and 203 (60.6%) thanks to the SoBeezy platform. Assistance with shopping and transportation, and home visits, were the 3 most frequently used services (n=54, 16.1%; n=51, 15.2%; and n=51, 15.2%, respectively), followed by homework/gardening (n=24, 7.2%), mobile hairdressing services (n=10, 3%), and other services (n=13, 3.9%). We then analyzed the characteristics of the service users compared with those who never used them (Table 2). The 2 groups did not differ in terms of age, living alone, number of interactions with BeeVA, and level of comfort with technologies. However, the SoBeezy service users all lived in Pessac. They were significantly more often women (n=23, 85%, vs n=4, 15%, men, *P*<.001), received home care services more often (n=13, 48%, vs n=14, 52%, *P*=.04), and tended to suffer more often from health problems (*P*=.18). See Table 2 for details. Among the 27 service beneficiaries, the level of satisfaction was very high (between 88% and 100%) for the following items: conditions of requests, quality of services, and delay of answers. It should be noted that 18 (72%) of 25 beneficiaries preferred using the telephone rather than BeeVA to request services.

**Table 2:** Description and comparison of participants’ characteristics according to the number of services received (n=69).

Characteristics		Service usage, n (%)	
		At least once (n=27)	Never (n=42)
**Age (years)**			
	62-81	14 (52)	22 (52)
	81-95	13 (48)	20 (48)
**Gender**			
	Man	4 (15)	12 (29)
	Woman	23 (85)	30 (71)
**Pilot city**			
	Pessac (urban area)	27 (100)	15 (36)
	St Jean de Luz (urban area)	0	14 (33)
	St Yrieix la Perche (rural area)	0	8 (19)
	Langon (rural area)	0	5 (12)
**Marital status**			
	In a relationship	8 (30)	12 (29)
	Living alone	19 (70)	30 (71)
**Recipient of home care services**			
	Yes	13 (48)	10 (24)
	No	14 (52)	32 (76)
**Member of an association**			
	Yes	10 (37)	22 (52)
	No	17 (63)	20 (48)
**Global health**			
	No self-reported problems	8 (30)	21 (50)
	Walking difficulty	5 (18)	8 (19)
	Other self-reported health problems	14 (52)	13 (31)
**Interaction with BeeVA (times/month)**			
	0-4	19 (70)	25 (60)
	>4	6 (22)	9 (21)
	Missing	2 (8)	8 (19)
**Comfort level with online technologies**			
	Comfortable	9 (33)	19 (45)
	Not comfortable	15 (56)	20 (48)
	Missing	3 (11)	3 (7)

### Usage and Acceptability Regarding BeeVA

In the whole sample (N=109), the median of monthly interactions with BeeVA was 1.5 (IQR 0.69-3.5), and for the sample of participants who completed the final experimentation (n=69), the median of monthly interaction was 1.6 (IQR 0.7-3.8).

Throughout the study, the participants interacted with BeeVA up to 23.7 times per month, with a median of 1.6 times per month. Those who interacted at least 4 times per month were considered as the highest users. Globally, the characteristics of the participants did not differ according to the level of interaction with BeeVA (Table 3). Nevertheless, we observed that the highest users were significantly younger (n=15, 75%, vs n=5, 25%), more often members of an association (n=12, 60%, vs n=8, 40%), and more likely to have walking and health problems (n=10, 25%, and n=10, 50%, respectively, vs n=5, 25%). The participants less comfortable with technology tended to interact more often with BeeVA than the others (n=13, 65%, vs n=30%).

Globally, 41 (63%) users had a positive opinion toward BeeVA, and the most positive dimensions of acceptability concerned usefulness (n=50, 77%), ease of use (n=53, 82%), and ergonomics/design (n=55, 85%). Reliability and usage behavior showed poorer results, with 49% (n=32) and 45% (n=29) positive opinions, respectively. Note that the usage behavior dimension only concerns the use of BeeVA options and does not include the use of services. Regarding items related to the acceptability of BeeVA, 48 (77%) of 62 users had a good opinion of voice usage to request services and options. In addition, even though not everyone used the services, two-thirds (n=44, 69%) of the participants considered BeeVA as a reassuring presence, with a slightly higher proportion among nonusers of the services (29/40, 73%, vs 15/24, 63%).

**Table 3:** Description and comparison of participants’ characteristics according to monthly interaction with BeeVA (n=59^a^).

Characteristics		Monthly interaction with BeeVA^b^		
		Lowest users (1st tertile; [0-1.05[ times/month; n=20), n (%)	Middle users (1st-2nd tertile; 1.5-2.8 times/month; n=19), n (%)	Highest users (>2nd tertile; >2.8 times/month; n=20), n (%)
**Age (years)**				
	62-81	7 (35)	9 (47)	15 (75)
	81-95	13 (65)	10 (53)	5 (25)
**Gender**				
	Man	3 (15)	5 (26)	5 (25)
	Woman	17 (85)	14 (74)	15 (75)
**Pilot city**				
	Pessac (urban area)	14 (70)	11 (58)	14 (70)
	St Jean de Luz (urban area)	3 (15)	6 (31)	4 (20)
	St Yrieix la Perche (rural area)	3 (15)	2 (11)	2 (10)
	Langon (rural area)	0	0	0
**Marital status**				
	In a relationship	7 (35)	7 (37)	5 (25)
	Living alone	13 (65)	12 (63)	15 (75)
**Recipient of home care services**				
	Yes	4 (20)	9 (47)	6 (30)
	No	16 (80)	10 (53)	14 (70)
**Member of an association**				
	Yes	8 (40)	8 (42)	12 (60)
	No	12 (60)	11 (58)	8 (40)
**Global health**				
	No self-reported problems	10 (50)	10 (53)	5 (25)
	Walking difficulty	2 (10)	3 (16)	5 (25)
	Other self-reported health problems	8 (40)	6 (32)	10 (50)
**Comfort level with online technologies**				
	Comfortable	11 (55)	8 (42)	6 (30)
	Not comfortable	8 (40)	7 (37)	13 (65)
	Missing	1 (5)	4 (21)	1 (5)

^a^Data on 10 participants were missing.

^b^Percentages can add up to more than 100 because of rounding.

To highlight the influence of users' characteristics on acceptability dimensions, each dimension was described by age, gender, living status, city, global health, level of comfort with technology, and statistics of monthly interactions with BeeVA and services (Table 4). The main differences in age and gender mainly concerned usefulness and design. Men perceived BeeVA as more useful and were more positive about the design than women (n=13, 26%, vs n=37, 74%, and n=14, 25%, vs n=41, 75%, respectively). The youngest users were less positive regarding the design than the oldest ones (n=26, 47%, vs n=29, 53%) but reported more frequently the usefulness of BeeVA (n=28, 56%, vs n=22, 44%). Participants living alone used BeeVA more frequently than people living with someone (n=24, 53%, used all or almost all options vs n=5, 25%, of the others). The participants who were less comfortable with technology used BeeVA more often and considered it reliable more frequently than people who were more comfortable. However, no differences were observed in usefulness (n=27, 82%, vs n=20, 74%). Among users who benefitted from the services and answered the acceptability questionnaire (n=25, 39%), there were no differences in perceived usefulness, ease of use, and ergonomics/design. Nevertheless, the service users used BeeVA more often (n=13, 45%, vs n=16, 55%) and reported reliability problems more frequently (n=18, 78%, vs n=5, 22%) than their counterparts (n=40, 62%) who never used the services but answered the acceptability questionnaire.

**Table 4:** Description of participants’ characteristics according to BeeVA acceptability dimensions (n=65^a^).^b^

Characteristics		Perceived usefulness, n (%)		Option usage behavior, n (%)			Ease of use, n (%)		Reliability, n (%)				Design and ergonomics, n (%)	
		Useful (n=50)	Mixed opinion/not useful (n=15)	0 (n=18)	1-2 (n=18)	3-4 (n=29)	Easy (n=53)	Mixed opinion/not easy (n=12)	Reliable (n=32)	Unreliable (n=23)	No opinion (n=10)	Positive (n=55)		Mixed opinion/negative (n=10)
**Age (years)**														
	62-81	28 (56)	6 (40)	8 (44)	11 (61)	15 (52)	27 (51)	7 (58)	19 (59)	11 (48)	4 (40)	26 (47)		8 (80)
	81-95	22 (44)	9 (60)	10 (56)	7 (39)	14 (48)	26 (49)	5 (42)	13 (41)	12 (52)	6 (60)	29 (53)		2 (20)
**Gender**														
	Man	13 (26)	2 (13)	3 (17)	4 (22)	8 (28)	12 (23)	3 (25)	8 (25)	6 (26)	1 (10)	14 (25)		1 (10)
	Woman	37 (74)	13 (87)	15 (83)	14 (78)	21 (72)	41 (77)	9 (75)	24 (75)	17 (74)	9 (90)	41 (75)		9 (90)
**Pilot city**														
	Pessac (urban area)	32 (64)	8 (53)	10 (56)	9 (50)	21 (72)	32 (60)	8 (67)	21 (66)	11 (48)	8 (80)	34 (62)		6 (60)
	St Jean de Luz (urban area)	8 (16)	5 (33)	3 (17)	8 (44)	2 (7)	10 (19)	3 (25)	4 (13)	8 (35)	1 (10)	10 (18)		3 (30)
	St Yrieix la Perche (rural area)	7 (14)	1 (7)	4 (22)	1 (6)	3 (10)	7 (13)	1 (8)	4 (13)	3 (13)	1 (10)	7 (13)		1 (10)
	Langon (rural area)	3 (6)	1 (7)	1 (6)	0	3 (10)	4 (8)	0	3 (9)	1 (4)	0	4 (7)		0
**Living status**														
	Not living alone	15 (30)	5 (33)	6 (33)	9 (50)	5 (17)	16 (30)	4 (33)	10 (31)	10 (43)	0	19 (35)		1 (10)
	Living alone	35 (70)	10 (67)	12 (67)	9 (50)	24 (83)	37 (70)	8 (67)	22 (69)	13 (57)	10 (100)	36 (65)		9 (90)
**Comfort level with online technologies**														
	Very comfortable	20 (40)	7 (47)	6 (33)	10 (56)	11 (38)	21 (40)	6 (50)	12 (38)	11 (48)	4 (40)	23 (42)		4 (40)
	Not comfortable	27 (54)	6 (40)	9 (50)	7 (39)	17 (59)	28 (53)	5 (42)	19 (59)	8 (35)	6 (60)	28 (51)		5 (50)
	Missing	3 (6)	2 (13)	3 (17)	1 (6)	1 (3)	4 (8)	1 (8)	1 (3)	4 (17)	0	4 (7)		1 (10)
**Number of services received**														
	At least 1	20 (40)	5 (33)	7 (39)	5 (28)	13 (45)	20 (38)	5 (42)	16 (50)	18 (78)	6 (60)	20 (36)		5 (50)
	0	30 (60)	10 (67)	11 (61)	13 (72)	16 (55)	33 (62)	7 (58)	16 (50)	5 (22)	4 (40)	35 (64)		5 (50)
**Global health**														
	No self-reported problems	19 (38)	10 (67)	8 (44)	6 (33)	15 (52)	23 (43)	6 (50)	14 (44)	12 (52)	3 (30)	26 (47)		3 (30)
	Walking difficulty	10 (20)	1 (7)	4 (22)	4 (22)	3 (10)	10 (19)	1 (8)	7 (22)	3 (13)	1 (10)	9 (16)		2 (20)
	Other self-reported health problems	21 (42)	4 (27)	6 (33)	8 (44)	11 (38)	20 (38)	5 (42)	11 (34)	8 (35)	6 (60)	20 (36)		5 (50)
**Monthly interaction with BeeVA (times)**														
	0-4	30 (60)	13 (87)	15 (83)	11 (61)	17 (59)	33 (62)	10 (83)	18 (56)	19 (83)	6 (60)	36 (65)		7 (70)
	>4	14 (28)	0	0	6 (33)	8 (28)	12 (23)	2 (17)	9 (28)	3 (13)	2 (20)	12 (22)		2 (20)
	Missing	6 (12)	2 (13)	3 (17)	1 (6)	4 (14)	8 (15)	0	5 (16)	1 (4)	2 (20)	7 (13)		1 (10)

^a^Data on 4 participants were missing.

^b^Percentages can add up to more or less than 100 because of rounding.

### Description of the Global Assessment of the Intervention

Of 65 participants, 14 (22%) had a positive opinion, 36 (55%) had a mixed opinion, and 15 (23%) had a negative one (Table 5).

Participants having a positive opinion were more often younger, were women, lived more often as couples, had poorer health (n=9, 64%, positive vs n=2, 14%, among those in good health), and were significantly more often users of the SoBeezy services (n=11, 79%, of them were positive vs only n=3, 21%, of the nonusers of the services, *P*=.002). However, the participants who found BeeVA adapted to older adults seemed to have a better opinion of the intervention (n=12, 86%, vs n=2, 14%). According to the experimental site, we observed that the participants of St Jean de Luz had the least positive opinion (n=6, 40%, were negative vs n=7, 47%, in Pessac and n=1, 7%, in St Yrieix la Perche). Finally, one-fourth of those who found the intervention useful had a positive perception of the intervention (vs none in the comparative group, *P*<.001).

**Table 5:** Description and comparison of participants’ characteristics according to the overall perception of the intervention (n=65^a^).^b^

Characteristics		Positive (n=14), n (%)	Mixed (n=36), n (%)	Negative (n=15), n (%)
**Age (years)**				
	62-81	11 (79)	17 (47)	6 (40)
	81-95	3 (21)	19 (53)	9 (60)
**Gender**				
	Man	2 (14)	11 (31)	2 (13)
	Woman	12 (86)	25 (69)	13 (87)
**Pilot city**				
	Pessac (urban area)	13 (93)	20 (56)	7 (47)
	St Jean de Luz (urban area)	0	7 (19)	6 (40)
	St Yrieix la Perche (rural area)	0	7 (19)	1 (7)
	Langon (rural area)	1 (7)	2 (6)	1 (7)
**Marital status**				
	In a relationship	6 (43)	10 (28)	4 (27)
	Living alone	8 (57)	26 (72)	11 (73)
**Global health**				
	No self-reported problems	2 (14)	19 (53)	8 (53)
	Walking difficulty	3 (21)	6 (17)	2 (13)
	Other self-reported health problems	9 (64)	11 (31)	5 (33)
**Comfort level with online technology**				
	Comfortable	5 (36)	16 (44)	6 (40)
	Not comfortable	8 (57)	18 (50)	7 (47)
	Missing	1 (7)	2 (6)	2 (13)
**Monthly interaction with BeeVA**				
	0-4	9 (64)	21 (58)	13 (87)
	>4	4 (29)	9 (25)	1 (7)
	Missing	1 (7)	6 (17)	1 (7)
**Service usage**				
	At least once	11 (79)	11 (31)	3 (20)
	Never	3 (21)	25 (69)	12 (80)
**BeeVA was a presence**				
	Yes	9 (64)	27 (75)	8 (53)
	No/no opinion	4 (29)	9 (25)	7 (47)
	Missing	1 (7)		
**BeeVA was adapted to older people**				
	Yes	12 (86)	25 (69)	7 (47)
	No/no opinion	2 (14)	11 (31)	8 (53)
**Perceived usefulness**				
	Useful	14 (100)	32 (89)	4 (27)
	Mixed opinion/not useful	0	4 (11)	11 (73)
**Reliability**				
	Reliable	10 (71)	18 (50)	4 (27)
	Unreliable	2 (14)	14 (39)	7 (47)
	No opinion	2 (14)	4 (11)	4 (27)
**Usage behavior (times)**				
	0	4 (29)	6 (17)	8 (53)
	1-2	3 (21)	11 (31)	4 (27)
	3-4	7 (50)	19 (53)	3 (20)
**Perceived ease of use**				
	Easy	11 (79)	31 (86)	11 (73)
	Mixed opinion/not easy	3 (21)	5 (14)	4 (27)
**Design and ergonomics**				
	Positive	12 (86)	31 (86)	12 (80)
	Mixed/negative opinion	2 (14)	5 (14)	3 (20)

^a^Data on 4 participants were missing.

^b^Percentages can add up to more or less than 100 because of rounding.

### Improvement Tracks

The 5 most popular options to be integrated into the future BeeVA were easy access to trusted professionals (50/63, 79%), communication about city events (42/65, 65%), late-night pharmacy (42/65, 65%), activity propositions tailored to their needs (40/65, 62%), and videoconferencing option (37/65, 57%); see Multimedia Appendix 2.

Regarding activity sharing, 28 (44%) of 63 participants were interested. The users expressed more interest in consulting the propositions (n=30, 48%) than to themselves propose an activity to the community (n=19, 30%). Among the users who expressed their motivations for shared activities (n=39, 57%), the 3 most frequent reasons were to meet people (n=29, 74%), to find a pastime (n=27, 69%), and to share a hobby with others (n=27, 69%). Sharing leisure activities interested 30 persons (81% of the sample), followed by physical activities (n=25, 68%) and cultural/touristic activities (n=24, 65%). In total, 29 (73%) of 40 participants were interested in using such an activity-sharing tool (Multimedia Appendix 3).

### Feedback From the SoBeezy Operational Team

First, the team reported real satisfaction and gratitude from the older participants who were supported by SoBeezy throughout this health crisis period. This particular sanitary context clearly hindered the deployment of the program (impossibility to propose the activity-sharing component, yet particularly expected by the users) and its functioning. In this context, the team raised the difficulty of relying on volunteers for good functioning of the platform (insufficient number and lack of reactivity when solicited for help). Regarding the users, the team emphasized the resistance to change (“I’ve always used my paper calendar on my fridge, I will not change my functioning,” “I have my own radio, I don’t need a new one”) and the inflexibility and intransigency of some (“I want my shopping at 2:00 p.m., 6:00 is too late”) and mentioned the individual barriers to using BeeVA (older age, depression, cognitive impairment, poor health, and severe reluctance to technology). The team also insisted on the fact that in this generation, it appeared difficult to ask for help (“I don't want to bother anyone about this, I'll manage it as I can,” “I've always managed my life by myself, I don't want to rely on someone else”). Nevertheless, the participants usually accepted the assistance when it was proposed by the team.

Moreover, most participants succeeded in using BeeVA after a minimal training program, but most of them also called the SoBeezy team over the phone for confirmation, which induced an unplanned workload for the staff. The team identified the main obstacle regarding technological aspects: the lack of reliability of the internet connection and the hardware (with a series of failures). This issue seriously disturbed the users, especially as they appeared rapidly overtaken in dealing with technological problems, even mild ones (eg, switching on the device or restarting it). In addition, the team underlined that the first version of BeeVA proposed too many features and options on its home screen, which reduced the readability of the services proposed. A clearer and simplified version was developed and quickly replaced the first one, with a substantial increase in the comfort of use expressed by the users. In addition, an avatar was also added to the home screen (Figure 2) and was appreciated. Finally, the team highlighted the importance of an efficient network of local partners, with a central role of the municipality (for identifying persons to equip and local partners).

## Discussion

### Principal Findings

The SoBeezy program is an innovative intervention aiming at promoting and fostering healthy aging by proposing to older adults concrete solutions to face daily life, tackle loneliness, promote social participation, and reduce the digital divide, thanks to a specific voice assistant. The experimentation, conducted in real-life conditions among older adults living in the community, was launched during the COVID-19 crisis in 4 different sites for 12 months.

In total, 109 older persons were equipped with BeeVA to use the SoBeezy platform. Among them, 32 left the experimentation before its end. The 3 main reasons for discontinued technology use were concordant to the literature [43,44], with technical problems, health problems, and no need for services. In total, 335 interventions were conducted, and almost 40% of the participants requested services. Nevertheless, three-quarters of the whole sample considered BeeVA a reassuring presence, and few participants had a negative opinion about the program (15 of 69). Among the users, BeeVA appeared easy to use (82%) and useful (77%) for older participants. Alleviating social isolation and loneliness was an important goal of the program. However, the pandemic context did not allow experimentation with the main way to tackle loneliness (ie, activity sharing). Our conclusions on this issue are consequently more limited than expected, and further research is needed. Nevertheless, this experimentation (particularly thanks to feedback from the field) confirmed that loneliness is a complex status, often associated with difficult life paths, particular personality trait, isolation, depression, and poor health [13]. Fighting loneliness in the older population requires time and human resources to establish a relationship of trust to allow a person to recover from settled loneliness. Technology alone scarcely appears to be a solution in this vulnerable population. Therefore, we think that such a program (the initial one including sharing activities) could be more relevant to prevent the occurrence of loneliness among older adults at greater risk than to “treat” loneliness when settled. These results could suggest that such devices and services could be useful to deploy among older adults, particularly in persons in the digital divide, with mobility restrictions, limitations in activities of daily living, a small social network size, geographically distant relatives, or living in rural areas [16,45-47].

This study faced obstacles related to the targeted population and technological aspects. Indeed, the appropriation of a device requires new ways to perform some activities and to change one’s habits (eg, vocal communication, online order, listening to the radio), which is known to become more difficult with aging [48-51]. Second, to ensure the follow-up of their requests, the users progressively tended to use the telephone (not intended for this use), a well-established and reassuring habit. We thus faced a need for an immediate response to their request [52]. As previously reported in another study [21], this behavior is consistent with the preference of older adults to use synchronous communications (eg, phone calls and instant messaging). The SoBeezy team also faced the difficulty for an older adult to verbalize the need for help, which can be explained by the fear of disturbing or by the refusal to rely on someone else to perform activities of daily living that they have always done by themselves. For some people, requesting a service can be seen as a marker of old age, inducing the vision of vulnerable older people [53].

Nevertheless, the participants who used the services provided by the SoBeezy program were satisfied with the services' quality and the interactions with the contributors. Another lesson learned from this experimentation was the technological intransigence of the older users. Indeed, there is a common belief that technology must be doing better than other traditional existing things, otherwise technology could not be perceived as useful, nor easy to use [39]. Each technological incident was consequently difficult to accept by the users, negatively impacting usage behavior, acceptance, and possible long-term adoption [39,54]. Regarding specifically BeeVA, it gained rather positive acceptability, but it was penalized by reliability issues, such as instability of the internet connection, which strongly disrupted interactions with the voice assistant [55]. In addition, despite our recommendations, many participants switched off the voice assistant instead of leaving it on standby, with some consequences on the functioning of the devices (BeeVA and 4G Wi-Fi). We also faced a series of simultaneous unexplained device failures that required replacing the devices.

Our experimentation also emphasized interesting levers for actions for the next deployment and future research. First, the perception of ease of use, an ergonomic design, and good reliability appear to be facilitators of good acceptability of BeeVA, assuming that individual step-by-step training is conducted. A simple user manual and a hotline phone number are provided (with the risks of drift, as previously mentioned) [31]. Among the technological levers for action, we observed the importance of a user-centered approach, which is essential to understand users' needs in terms of technological skills and psychological characteristics (apprehension, reluctance, technophobia). A simplification of the steps to achieve the expected results is also essential for device appropriation, since older adults can be interested in technological devices. However, they can be discouraged when sophisticated computerized devices replace simpler ones, which are easier to use for them [40,56]. Moreover, the social environment may also play an important role in accepting the technology; the relatives could play the role of a mediator with a positive social pressure for the appropriation of new technology [21,46,53,57] and compensate for the prior lack of digital literacy [21]. As suggested by our study, the participants living with their partners tended to be more positive about the SoBeezy intervention and found BeeVA to be more reliable than people living alone. We also clearly identified important differences between the pilot sites, the program being more efficient when the local partnership was highly involved in the program, with a direct impact on the satisfaction of the users (46% had a negative opinion about the program in one site vs 13% in another). This result underlines the importance of solid partnerships with local actors, particularly for the diffusion and appropriation of the technology, the identification of available resources in a territory, and the identification of the “invisible” of the society (ie, isolated persons, often far from social and medical care systems, despite greater health or social problems) [58]. Taken together, our results converge with the conceptual framework developed by the Center for Research and Education on Aging and Technology Enhancement (CREATE). This framework highlights the importance of considering factors related to the microscopic level (eg, the capacity of the person, the services, and the interaction with the technology) and those related to the macroscopic level (eg, the social environment and support of the person) [45].

Finally, tackling loneliness is 1 of the main objectives of the SoBeezy program. Unfortunately, its deployment has been greatly affected by the pandemic context, and we could not particularly experiment with the main component targeting loneliness (ie, the activity-sharing component). However, it is also well known that isolated people and those suffering from loneliness are more likely to refuse help and assistance, particularly when affected by the digital divide [34]. Nevertheless, the PRISM platform [31,32] reported a significant decrease in the feeling of loneliness and an increase in perceived social support and well-being among participants living alone and a good appropriation of the technology.

### Strengths and Limitations

In experimenting on technologies for older people, the number of participants equipped with BeeVA (more than 100 older adults, including those in the digital divide) represents 1 of the strengths of the study. Second, the SoBeezy services, being free of charge, guaranteed wide and egalitarian access [59]. Moreover, the participants could choose the services that seemed appropriate to them at their convenience and according to their own needs. This choice ensured a good level of agentivity and self-determination in the appropriation of the SoBeezy platform [60]. Our results also showed the importance of the user-centered approach, which allows adapting a device and contributing to better learning and appropriation by the aging population [37,56].

However, our study also has some limitations. For even easier use of the device, natural language understanding may be improved using artificial intelligence methods. Due to the sanitary context, we could not experiment the sharing-activity component, whereas it was the first objective of this program to combat social isolation and loneliness. Moreover, the evaluation of the program was limited by a potential selection bias; the final assessment was not available for the participants who left the experimentation early—yet more likely to be unfavorable to the intervention. It is important to emphasize that our sample size was probably adequate to identify large significant effects but insufficient to detect medium or small effects. Therefore, we only reported the *P* values of significant relevant effects. Regarding the usage frequency of BeeVA, we did not have access to detailed individual data, such as time of usage of an option or an app, or accidental clicks, that represents a limitation of the data on the interactions with the device. We did not assess perceived loneliness but only the living status (living alone vs not living alone). Finally, the interviewers reported a risk of social desirability bias, the participants being particularly grateful for the support provided during the COVID-19 crisis.

### Perspectives: Guidelines for Improved Deployment of SoBeezy

Several improvements were conducted during the experimentation. First, the final interviews allowed the older users to suggest new services or options that could interest them, such as easy access to trusted professionals or communication about city events. These services should be proposed in the next deployment of the SoBeezy program.

Second, our results also suggested the potential benefits of a close network of older users to improve the confidence that users have in services and activity sharing (see Multimedia Appendix 3 presenting the potential areas of improvement). Therefore, we suggest relying more on older adults already in a community, such as the seniors' clubs or independent living housing [36].

Regarding the personalization of the device, it could be interesting to propose to BeeVA users a mixed mode (voice and touch) and several versions of SoBeezy, for smartphones, tablets, or computers (ie, responsive design), and give them a choice to fit their preferences and habits.

Regarding the training phase, in addition to written instructions (hard-copy format), it could be interesting to provide interactive and personalized training sessions (eg, through games and videos of uses adapted to the level of the participants). These sessions would allow older people to better accept the technological solutions and help both the acceptance of asynchronous communications and a better use of the functionalities available on the device [31]. These individual and collective training sessions could not be implemented due to the COVID-19 context.

Finally, from a technical point of view, it could be judicious to record, for example, interaction errors under the use of some services to improve device reliability. For service providers on the platform, it could be also useful to give them feedback on the usage (number of users, frequency and type of use) to improve the proposed services.

### Conclusion

This multisite study conducted in real-life conditions with more than 100 older adults living in the community provides enlightening results for the reality on the ground, specifically when we are interested in digital tools designed for the aging population. The context of the COVID-19 epidemic was, on the one hand, favorable in the light of the massive needs of the older adults during this crisis but, on the other hand, particularly limiting due to the sanitary measures that clearly affected the program. The experimentation overall showed a positive acceptability of the voice assistant (ie, perceived usefulness and ease of use) and a high level of satisfaction of the participants who used SoBeezy. Nevertheless, our findings also highlighted the issues of resistance to change, difficulties for the users in asking for help, and difficulties met to efficiently tackle chronic settled loneliness using ICT. The SoBeezy program could be a way to improve the autonomy and well-being of older adults and their families. The next step will be the experimentation with the activity-sharing component that could not be tested due to the COVID-19 context.

## Appendix

Multimedia Appendix 1 Derivation of the variable measuring BeeVA acceptability.

Multimedia Appendix 2 Distribution of opinions on possible functions to be integrated into BeeVA among participants surveyed.

Multimedia Appendix 3 Repartition of the areas for improvement and reorientation of BeeVA, according to overall BeeVA acceptability.

## Abbreviations

ICT: information communication technology
PRISM: Personal Reminder Information and Social Management
SNS: social network services
SOS: social online services

## Data Availability

The data sets generated and analyzed during the study are available from the corresponding author upon reasonable request.
