# Supplementary figures and images for: Lessons Learned From the SoBeezy Program for Older Adults During the COVID-19 Pandemic: Experimentation and Evaluation
Source: JMIR Form Res. 2022 Nov 24;6(11):e39185. doi: 10.2196/39185 (PMC9697092; doi:10.2196/39185)

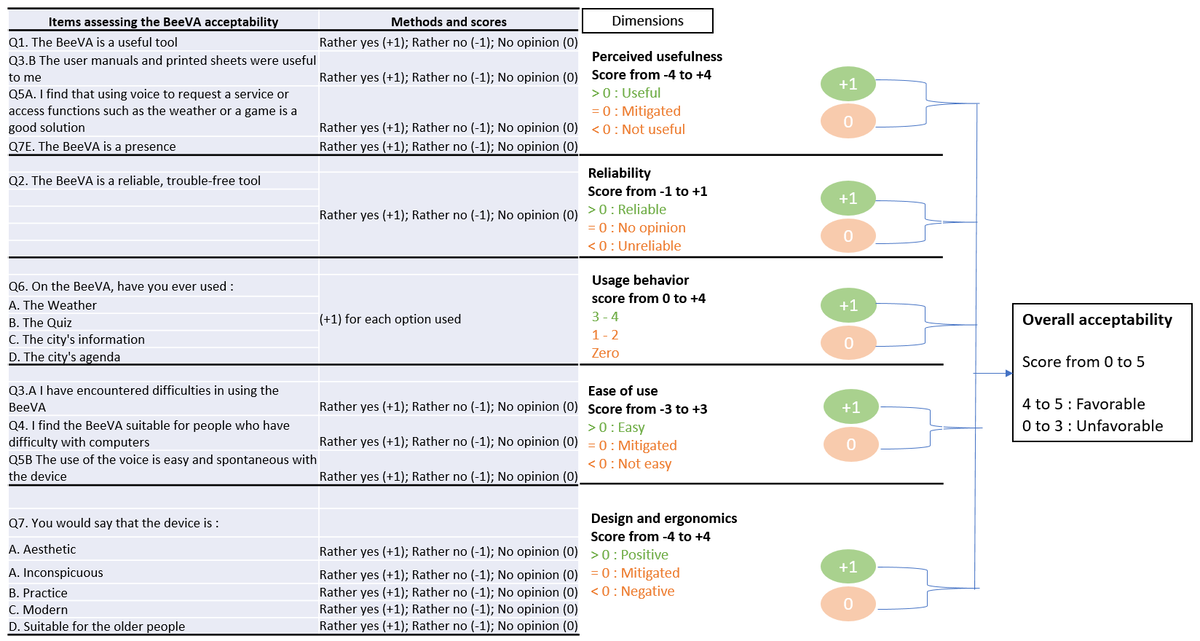

Supplement: Multimedia Appendix 1 [file formative_v6i11e39185_app1.png]

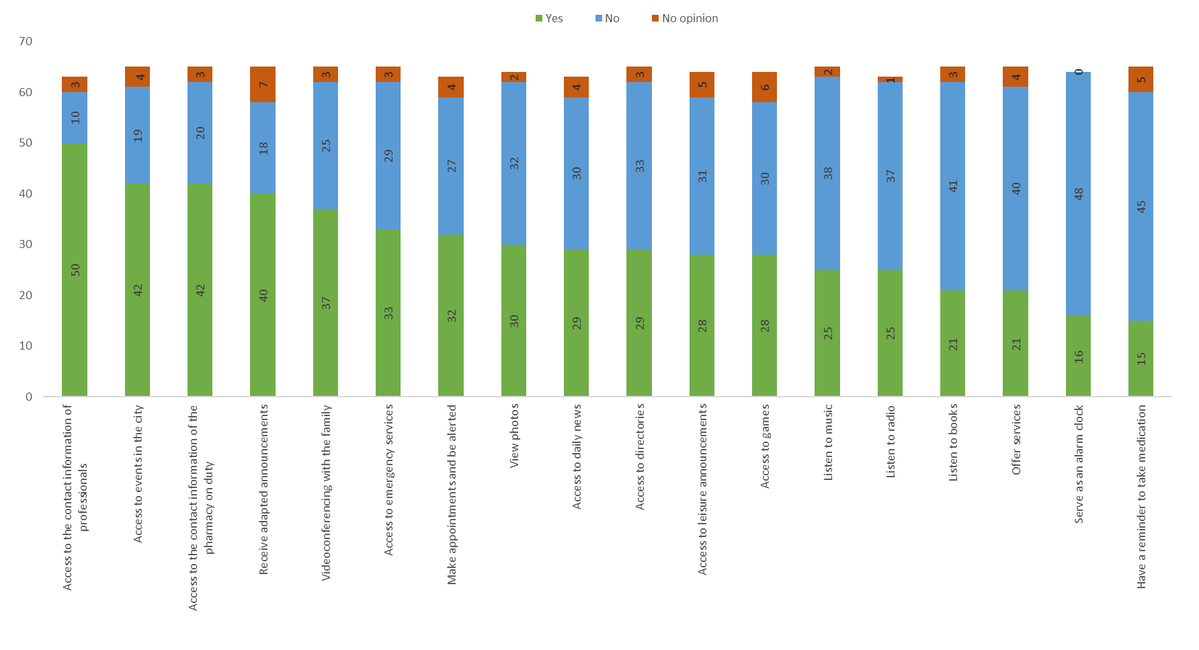

Supplement: Multimedia Appendix 2 [file formative_v6i11e39185_app2.png]
