# Supplementary material for: Lessons Learned From the SoBeezy Program for Older Adults During the COVID-19 Pandemic: Experimentation and Evaluation
Source: JMIR Form Res. 2022 Nov 24;6(11):e39185. doi: 10.2196/39185 (PMC9697092; doi:10.2196/39185)
Supplement: Multimedia Appendix 3 [file formative_v6i11e39185_app3.pdf]

Ideas for improvement and reorientation of the BeeVA	Overall BeeVA acceptability						
	Total		Favorable (n=41)		Unfavorable (n=24)		MD*
	n	%	n	%	n	%	
**Interest in relationship device**							6
Yes (Moderately to very interested)	28	44.4	19	48.7	9	37.5	
**To suggest leisure activities on the device**							6
Oui	19	30.2	12	30.8	7	29.2	
Non	44	69.8	27	69.2	17	70.8	
**Learn about leisure activities on the device**							6
Oui	30	47.6	19	48.7	11	45.8	
Non	33	52.4	20	51.3	13	54.2	
**Would you be willing to write a leisure activity and/or outing**							
**To keep me busy**							35
Yes	14	41.2	9	40.9	5	41.7	
No	20	58.8	13	59.1	7	58.3	
**To meet people**							34
Yes	17	48.6	11	47.8	6	50.0	
No	18	51.4	12	52.2	6	50.0	
**To share my hobby**							34
Yes	19	54.3	13	56.5	6	50.0	
No	16	45.7	10	43.5	6	50.0	
**To join a community**							34
Yes	12	34.3	6	26.1	6	50.0	
No	23	65.7	17	73.9	6	50.0	
**To boost a neighborhood**							34
Yes	9	25.7	5	21.7	4	33.3	
No	26	74.3	18	78.3	8	66.7	
**Would you be willing to read a leisure activity and/or outings**							
**To keep me busy**							30
Yes	27	69.2	18	72.0	9	64.3	
No	12	30.8	7	28.0	5	35.7	
**To meet people**							30
Yes	29	74.4	18	72.0	11	78.6	
No	10	25.6	7	28.0	3	21.4	
**To share my hobby**							30
Yes	27	69.2	18	72.0	9	64.3	
No	12	30.8	7	28.0	5	35.7	
**To join a community**							31
Yes	17	44.7	10	41.7	7	50.0	
No	21	55.3	14	58.3	7	50.0	
**To boost a neighborhood**							30
Yes	12	30.8	7	28.0	5	35.7	
No	27	69.2	18	72.0	9	64.3	

Ideas for improvement and reorientation of the BeeVA	Overall BeeVA acceptability						
	Total		Favorable (n=41)		Unfavorable (n=24)		MD*
	n	%	n	%	n	%	
**Importance of the some criteria**							
**People recommended by other users**							32
Important	34	91.9	23	95.8	11	84.6	
Not important	3	8.1	1	4.2	2	15.4	
**Secure tool**							32
Important	37	100.0	24	100.0	13	100.0	
Not important	0	0.0	0	0.0	0	0.0	
**People I know**							32
Important	35	94.6	24	100.0	11	84.6	
Not important	2	5.4	0	0.0	2	15.4	
**Someone who lives near you**							32
Important	27	73.0	20	83.3	7	53.8	
Not important	10	27.0	4	16.7	6	46.2	
**Knowing the availability of each other users**							32
Important	17	45.9	12	50.0	5	38.5	
Not important	20	54.1	12	50.0	8	61.5	
**Proposed activities and (or) outings that would be of interest**							
**Tourist and cultural outings**							32
Yes	24	64.9	16	66.7	8	61.5	
No	13	35.1	8	33.3	5	38.5	
**Physical activities**							32
Yes	25	67.6	17	70.8	8	61.5	
No	12	32.4	7	29.2	5	38.5	
**Leisure activities**							32
Yes	30	81.1	19	79.2	11	84.6	
No	7	18.9	5	20.8	2	15.4	
**What device would you use to propose or participate in an**							
**Via a call platform**							31
Yes	20	52.6	14	58.3	6	42.9	
No	18	47.4	10	41.7	8	57.1	
**Via a smartphone application**							31
Yes	23	60.5	15	62.5	8	57.1	
No	15	39.5	9	37.5	6	42.9	
**Via tablets connected to the internet**					31		31
Yes	28	73.7	19	79.2	9	64.3	
No	10	26.3	5	20.8	5	35.7	
**Via website**							31
Yes	24	63.2	18	75.0	6	42.9	
No	14	36.8	6	25.0	8	57.1	
**How do you find the concept?**							29
Moderately to very interesting	34	85.0	23	88.5	11	78.6	
Little or not interesting	6	15.0	3	11.5	3	21.4	
**Would you be willing to use the device?**							29
Yes	29	72.5	22	84.6	7	50.0	
No	11	27.5	4	15.4	7	50.0	

* missing data
